# Rapid forward-in-time simulation at the chromosome and genome level

**DOI:** 10.1186/1471-2105-14-216

**Published:** 2013-07-09

**Authors:** Andre J Aberer, Alexandros Stamatakis

**Affiliations:** 1 The Exelixis Lab, Scientific Computing Group, Heidelberg Institute for Theoretical Studies, Schloss-Wolfsbrunnenweg 35, Heidelberg D-69118, Germany

**Keywords:** Population genetics, Forward-in-time simulation, Fisher-Wright model, Algorithm, Software, Natural selection

## Abstract

**Background:**

In population genetics, simulation is a fundamental tool for analyzing how basic evolutionary forces such as natural selection, recombination, and mutation shape the genetic landscape of a population. Forward simulation represents the most powerful, but, at the same time, most compute-intensive approach for simulating the genetic material of a population.

**Results:**

We introduce AnA-FiTS, a highly optimized forward simulation software, that is up to two orders of magnitude faster than current state-of-the-art software. In addition, we present a novel algorithm that further improves runtimes by up to an additional order of magnitude, for simulations where a fraction of the mutations is neutral (e.g., only 10% of mutations have an effect on fitness). Apart from simulated sequences, our tool also generates a graph structure that depicts the complete observable history of neutral mutations.

**Conclusions:**

The substantial performance improvements allow for conducting forward simulations at the chromosome and genome level. The graph structure generated by our algorithm can give rise to novel approaches for visualizing and analyzing the output of forward simulations.

## Background

The field of population genetics strives to determine, how elementary evolutionary forces (i.e., natural selection, recombination, or random genetic drift) shape the genetic landscape within population of a species. As for related areas in evolutionary biology, rapid advances in next-generation sequencing technology (e.g., [1]) have transformed the field with the completion of the first phase of the 1000 genome project [2] representing a recent highlight. Thus, population genetics is rapidly transforming into a data-driven science [3], while many methods and tools are not up to the challenge yet [4] with respect to scalability and efficiency.

Because of the complex processes in population genetics, the inference machinery for analyzing the properties of a population is often limited and needs to be complemented by simulations. Some of the most common use cases of simulation software in population genetics are (i) the verification of novel methods/models [5,6] and (ii) the generation of datasets for assessing how using a different model affects the properties of of a population. A promising, but computationally particularly expensive approach is to use simulations for inferring specific properties (e.g., demography [7]) of a given data sample, as implemented, for instance by using approximate Bayesian computation techniques [8]. For an overview of available software and applications, see [9].

Forward-in-Time simulation represents a powerful approach to simulating the evolutionary processes that act on genomic regions (see review by [10]). Essentially, sequences are represented *in silico* and the basic evolutionary operations/events (e.g., mutation, selection, and recombination) are explicitly applied to each simulated individual, on a generation-by-generation basis. The inferred, simulated sequences represent the exact outcome of the underlying/assumed evolutionary process. However, this high accuracy comes at a high computational cost. Execution times are quadratic with respect to the number of individuals, because the number of simulated generations needs to be increased with growing population size. To obtain accurate results, the size of the population that needs to be simulated must be chosen realistically. However, realistic population sizes range between a computationally already challenging 10,400 individuals in humans to an entirely prohibitive number of 1,150,000 individuals in some species of *Drosophila melanogaster* or 25,000,000 in *Escherichia coli*[11].

These excessive computational requirements explain the popularity of approximate coalescent (backward) simulations [12,13] that are substantially faster. The coalescent-based approach is faster because only genomic samples that survived until the present are simulated *backward* in time. A substantial drawback of coalescent simulations is that, natural selection can only be incorporated into the model by means of a single mutation [14,15].

To date, only few attempts have been made to improve the performance of forward simulators using optimization techniques from computer science and via low-level technical improvements. Chadeau-Hyam *et al.* developed a strategy to re-scale simulation parameters such as to decrease the effective population size that needs to be simulated [16]. However, this shortcut induces a decrease in accuracy (see [17]). Forwsim[18] (later extended by [19]) implements a forward-algorithm that regularly simulates a small user-defined set of mutations under selection, forward in time. The neutral part of each haplotype is simulated separately and executed with a delay of 8 generations. Thereby, one can circumvent simulating haplotypes, if no ancestral material has survived until the present. Distinguishing between neutral and non-neutral mutations for accelerating forward simulations represents a natural implementation choice, specifically since it is assumed that the majority of mutations is effectively neutral [20].

Finally, SFS_CODE[21] has become a *state-of-the-art* forward simulator, because of an efficient implementation in C coupled with a plethora of simulation features and parameters.

Here, we describe design as well as optimization techniques that allow for substantially more efficient forward-in-time simulations. We introduce a novel algorithm, for the *a posteriori* simulation of neutral mutations. In addition to runtime gains, our algorithm generates a data structure that depicts the entire observable history of each individual sequence, a feature that –to the best of our knowledge– is unique among simulation software. We make available a corresponding software package, the *Ancestry-Aware Forward-in-Time Simulator* (AnA-FiTS, available under GNU GPL at our project webpage: http://www.exelixis-lab.org/aberer/anafits/index.html), a C++ implementation that can handle challenging post-genomic datasets for population genetics analyses.

## Methods

In the following, we describe our algorithm for ancestry-based simulation of neutral mutations. Subsequently, we discuss implementation and optimization issues.

### Algorithm for simulating neutral mutations

For forward simulation of a single generation of a population with effective population size *N*_*e*_, a scaled mutation rate of *θ*=4*N*_*e*_*μ* (with *μ* being the per-base mutation rate), the scaled recombination rate *ρ*=4*N*_*e*_*r* (with *r* being the per-base recombination rate), the basic simulation steps are: (i) sample diploid individuals by their fitness, (ii) determine the number of recombination events (Poisson random number drawn from Poi(*ρ*)) and create recombinants, (iii) determine the number of non-neutral mutations (Poisson random number drawn from Poi(*θ*)) and mutate sequences, (iv) recalculate the fitness values for each individual.

Since neutral mutations, by definition, do not affect the survival probability of an individual (resp. the sampling probability), we do not need to simulate neutral mutations forward in time. This observation was first explored in forwsim[18], where the simulation of the neutral part of a sequence was delayed for a small number of generations. Thereby, the actual calculations required for this part of the simulation can be discarded, if the haplotype under consideration did not survive (because of drift or selection) until the present. The authors demonstrated that, the simulation of neutral sequences can be accelerated by a factor of two to five by optimally choosing this so-called look-ahead parameter, that is, the number of generations, by which the simulation of neutral mutations is delayed.

**Figure 1:**
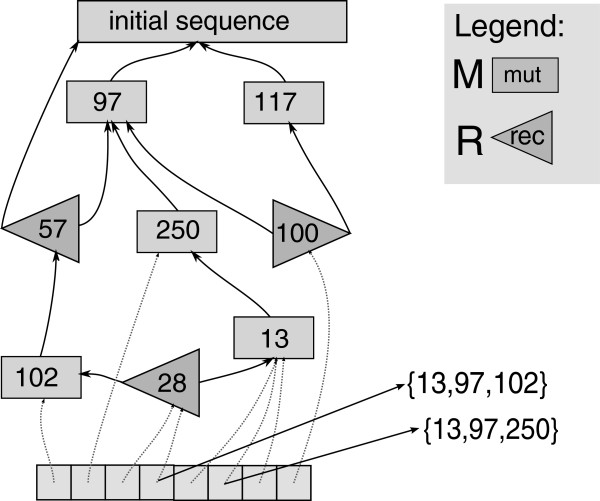
**Backward event graph.** The graph consists of M-nodes (mutations) and R-nodes (recombinations). Edges represent ancestral relationships, numbers stand for breakpoints (in R-nodes) or mutated sequence position (in M-nodes). R-nodes point toward the donor of the sequence at the downstream side of the breakpoint. Two example sequences are extracted from the graph (lower right).

In AnA-FiTS, we deploy a different strategy: neutral mutations are not simulated forward in time at all. Instead, we keep track of the entire ancestry (and all recombination events) of all surviving individuals (potentially for several thousand generations), create a graph structure (see Figure 1), and only extract the neutral part of all sequences from this graph structure once the forward simulation has been completed. In other words, we retain all information accumulated during the forward simulation phase, exactly determine all parts of sequences, where neutral mutations could have occurred and finally insert neutral mutations into the sequence *a posteriori*. Note that, in analogy to forwsim and in contrast to coalescent simulations, our algorithm is equivalent to simulating all neutral mutations forward in time. Thereby, we can guarantee simulation accuracy, which is one of the key advantages of forward simulations.

#### Simulating surviving neutral mutations

During *l* forward-simulation generations (typically *l*:=10*N*_*e*_ for diploid organisms), AnA-FiTS annotates an ancestry $A_{l}=\{a_{1},a_{2},\ldots a_{l}\}$, where *a*_*i*_ is a surjective function that maps the individual haplotypes in generation *i* to the haplotypes of the preceding generation *i*−1. In the following, we outline this procedure for a single locus/chromosome. The extension to multiple loci is described in the implementation section. The variable *a*_*i*_ is defined as *a*_*i*_:*h*_*i*,*j*_→*h*_*i*−1,*l*_, where *h*_*i*,*j*_ is the haplotype with index *j* in generation *i*, that is mapped to its parental haplotype with index *l* in generation *i*−1. Haplotype indices are ordered, such that adjacent pairs that start with an even index represent the two homologous versions of each locus for diploid organisms (i.e., (*h*_*i*,0_,*h*_*i*,1_) or (*h*_*i*,2_,*h*_*i*,3_) represent an individual).

As mentioned before, the algorithm also keeps track of all recombination events (potentially multiple recombinations per sequence and per generation) that occurred during the forward phase. For each event, we store the absolute sequence position, the haplotype index, and the generation number. For a sequence of length *L*, the information in $A_{l}$ and the corresponding set of recombination events can be used to determine (via back-tracking) the regions of ancestral haplotypes that survived until the present. Only mutations that occurred within these regions are observable in the present. In the first step, our algorithm determines haplotypes in ancestral generations (referred to as *survivors*) that contributed material to the present generation (denoted *surviving regions*). For these regions, we simulate neutral mutations and identify those recombination events that contributed to mutations in the present haplotype instances.

The algorithm starts at the present generation and initializes surviving regions for each haplotype with a maximum interval of [ 0,*L*], where *L* is the sequence length. Regions for ancestral haplotypes are initialized by the tuple (*∞*,0). Note that, the surviving regions of a haplotype instance can be fragmented/scattered into many segments by recombinations. To economize on runtime and memory, we only keep track of the start position of the very first segment *r*_*S*_ and the end position of the very last segment *r*_*E*_. By using this approximate surviving ’super-’region *R*=(*r*_*S*_,*r*_*E*_), we overestimate the size of the actual surviving region. We correct for this in a later stage of the algorithm, when sequences are extracted from the graph structure. Finally, to determine the surviving haplotype region in generation *i*−1, we iterate over all survivors $S_{i}$ of generation *i* starting with haplotypes contained in the present generation. For each surviving haplotype $h_{i,l}\in S_{i}$ with region (*r*_*S*_,*r*_*E*_), we propagate the surviving regions as described in the following algorithm to the members of the preceding generation. Let *h*_*i*−1,*k*_ with region $\left(r_{S}^{\prime },r_{E}^{\prime }\right)$ be the parent haplotype and *h*_*i*−1,*k*+1_ with region $\left(r_{S}^{*},r_{E}^{*}\right)$ be the homologous haplotype in the corresponding parent individual: 

For simplicity, the algorithm described above assumes that the recombinant emerged from at most one recombination event. For multiple recombination events per recombinant, the algorithm has to be adapted, such that surviving regions of parental haplotypes are extended until the next recombination breakpoint. This has also been implemented in the AnA-FiTS software.

During the procedure described above, we also determine the set of survivors $S_{i-1}$ from the preceding generation. In the case that a recombination event does not split the surviving region (*r*_*S*_,*r*_*E*_), we can simply ignore it, if the following property does not hold: assume, haplotype *h*_*i*,*l*_ is mapped to *h*_*i*−1,*k*_ with surviving region (*r*_*S*_,*r*_*E*_) and *h*_*i*,*l*_ is a recombinant with a breakpoint *b*<*r*_*S*_. This means that, *h*_*i*,*l*_ does not inherit mutations from *h*_*i*−1,*k*_, but instead from the homologous haplotype *h*_*i*−1,*k*+1_ it recombined with. If we can not ignore the recombination, we create a node instance for the graph that will be constructed in the next step. The survivors of each generation are stored for further processing in subsequent steps.

**Figure 2:**
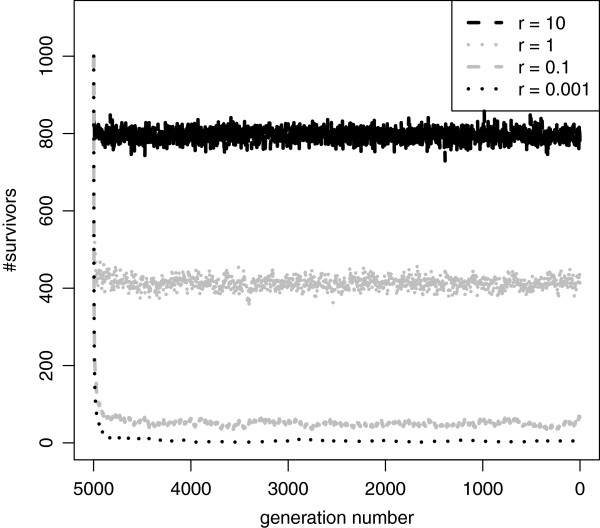
**Surviving haplotypes per generation.** Number of haplotypes that may contribute genetic material to the present generation (*survivors*) for various per-sequence / per-generation recombination rates *r*∈{10,1,0.1,0.001}, *N*_*e*_=500 and a sequence length of 10^7^ nucleotides.

Once survivors and surviving regions for a generation *i* have been determined, we can then simulate the neutral mutations that occurred in this generation *and* that are still observable in the present generation. Thus, the expected number of neutral mutations is reduced by a factor of $F=\frac{\left|S_{i}\right|}{2\cdot N_{e}}$. Furthermore, if a neutral mutation occurs outside the estimated surviving region, it can simply be ignored. As shown in Figure 2, depending on the recombination rate *r*, the number of haplotypes that contribute genetic material to the present generation quickly converges and may cause values of *F* to become small. Note that, we expect the algorithm to become inefficient for exceptionally high recombination rates, such as *r*:=10 (i.e., 10 recombinations per individual and per generation).

#### Graph construction

In the algorithm step described above, we determined survivors $S_{i}$ for each generation *i*, the set of relevant recombinations (stored as so-called *R-nodes*), and the set of relevant neutral mutations (so-called *M-nodes*). For all nodes, we store the generation of origin and the corresponding haplotype index *h*_*i*,*l*_. The goal of the procedure we describe here, is to construct a graph (the *Backward Event Graph* BEG, see Figure 1 for an example). The BEG is a directed acyclic graph with two types of nodes: (i) M-nodes (for mutations) with out-degree 1 and (ii) R-nodes (for recombinations) with out-degree 2. Thus, each node in the graph represents the state of an ancestral haplotype that reflects the history of all mutation or recombination events experienced by its ancestors. A directed edge connecting node *n*_1_ to node *n*_2_ indicates that *n*_2_ emerged from *n*_1_ after mutation or recombination.

**Figure 3:**
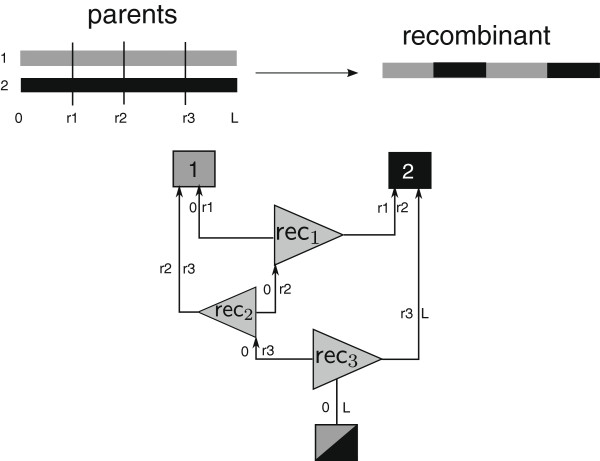
**Information flow in the BEG graph.** An example of the information flow (i.e., regions that potentially contain ancestrial mutations) for a recombinant that underwent three recombination events. Each line is labeled with the start (*left* of edge) and end (*right* of edge) of regions that survive.

Given this information, the graph can be constructed forward in time (starting with generation 0) using an array *A*_*i*_ for the current generation and an array *A*_*i*−1_ for the preceding generation *i*−1 that contains node references/pointers. Array *A*_*i*_ is populated by carrying over/propagating references from *A*_*i*−1_ according to the survival information stored in $S_{i-1}$. If a surviving haplotype of generation *i* underwent recombination or mutation, the respective R- or M-node *n*_new_ is used to replace the node *n*_old_ that was propagated and an edge is created between *n*_new_ and *n*_old_. We also create an additional edge to the haplotype which is homologous to *n*_old_ for R-nodes (recombination). Figure 3 provides a complex example, where a recombinant emerges from three recombination events that originated at two homologous ancestral haplotypes 1 and 2.

#### Extraction of sequences

The algorithm described above constructs the BEG and an array of haplotype states *n*_*i*_ (references to nodes in the BEG), that survived until the present. In the final step, we extract the exact sequence for each node *n*_*i*_ from the BEG by recursively traversing the graph starting at *n*_*i*_, while keeping track of the region of interest which is segmented by each recombination event which will be encountered. Since we execute the backtracking procedure for each node *n*_*i*_ individually, (in contrast to the algorithm description) the exact borders of all segments of the surviving region for this node are known at any given point in time. Thus, we can ignore all mutation events that did not occur within the surviving region, but were inserted into the graph because of overestimation or because this mutation forms part of the surviving region of another haplotype.

In practice, the above extraction procedure is inefficient. As a consequence, the sequence reconstruction step dominates execution times. Consider two haplotype states *n*_1_ and *n*_2_ that have survived, where *n*_2_ is an M-node with ancestor *n*_1_. For both nodes, we have to traverse all ancestral nodes of *n*_1_ and *n*_2_ to obtain the two corresponding sequences *s*_1_ and *s*_2_. To accelerate this process, it suffices to create sequence *s*_1_ via backtracing starting at *n*_1_ and to generate *s*_2_ as a copy of *s*_1_ and then simply add the mutation events induced by node *n*_2_ in the end. In the following, we explain, how the computational cost of sequence extraction can be reduced.

Prior to the actual sequence extraction step, the graph is traversed twice, starting with each haplotype *n*_*i*_ that survived until the present. In the first traversal, we approximate the surviving region for each node as described in the algorithm. Thus, if we decide to explicitly determine and store the sequence of a node, we already know the maximum size of the region of interest beforehand.

In a second traversal that starts with each haplotype in the present, we determine for which nodes intermediate sequences should be created. In other words, the surviving mutations are represented explicitly in this node. During these traversals, we actually stop a traversal, if the traversed node has already been visited by another traversal. For each node, we also store the number *v* of times the node has been visited. We refer to nodes with *v*≥2 as *coalescent nodes*, since ancestral material (resp. the surviving regions) of two lineages coalesces in these nodes. Finally, we determine the distribution of *v* and the 5% nodes with highest *v* are represented explicitly, while all remaining nodes will be traversed several times during sequence extraction.

Then, the sequences can be extracted via a final full traversal. The 5% cut-off (a tuning parameter of the algorithm) was determined empirically. It yields good results with respect to balancing runtime versus memory requirements. Note that, if every coalescent node (*v*≥2) is represented explicitly, memory requirements would become prohibitive for whole-chromosome simulations with 10,000 individuals. In general, the 5% cut-off proves to be sufficient to attain a substantial decrease of the traversal cost for sequence extraction.

### Implementation and optimization

The ancestry-based algorithm for simulating neutral mutations as discussed in the previous section has specifically been developed to improve runtimes of forward-simulations. In the following, we discuss low-level implementation issues and optimization techniques of AnA-FiTS.

#### Memory requirements of ancestry and BEG

For the majority of possible AnA-FiTS invocations with respect to user parameter combinations, storing the ancestry $A_{l}$ requires the largest amount of memory. Depending on the effective population size *N*_*e*_, we need to store ancestry information for 10·*N*_*e*_^2^ haplotypes. If this exceeds the amount of available memory, the user can set a command line flag such that the memory utilization of the ancestry does not exceed a user-specified soft limit (see below). AnA-FiTS then tries to split up the forward simulation into sections (a number of generations that is as large as the user parameter permits) and updates the graph after each section leading to increased runtimes. In some instances the graph representation itself can become at least as memory intensive as the ancestry. Furthermore, for large graphs, the explicit bit vector representation of intermediate sequences may further increase memory requirements. Therefore, currently no hard upper memory utilization limit can be imposed on AnA-FiTS runs.

The ancestry $A_{l}$ only works correctly for a single locus. For multiple unlinked loci (e.g., chromosomes), we map each individual to its two parent individuals instead of mapping it to individual haplotypes. For this, we use a per-chromosome bit vector to keep track of which of the two possible homologous haplotypes was inherited from the respective parent. Apart from their high memory-efficiency, bit vectors can also be efficiently initialized with random bits using a random number generator (i.e., each bit represents a choice as to which haplotype was inherited by an individual).

To avoid unnecessary and inefficient frequent memory allocations, the entire ancestry (an array of arrays) and the array of survivors are allocated as single, monolithic blocks. For the ancestry, we dynamically determine the minimal number of bytes that is required for storing a generation as a function of the population size of the preceding generation (e.g., if the previous generation contained 250 individuals, a 8-bit unsigned char is sufficient). Since, under most simulation scenarios, *N*_*e*_ does not exceed 65,536 individuals, it is mostly sufficient to use the unsigned short integer (=2 bytes) datatype for saving memory.

Note that, beside all efforts to keep the memory consumption of AnA-FiTS as low as possible, the entire ancestry (or –as described above– part of it) is kept in main memory during simulation. This necessarily means that AnA-FiTS exhibits higher memory requirements than comparable programs.

#### Random number generation

High quality (pseudo-)random number generators are essential for simulation tools. To date, the Mersenne twister algorithm [22] is considered as state-of-the-art in pseudo-random number generation (PRNG). A SIMD-based version of the Mersenne twister (SFMT) [23] employs Streaming *Single instruction, multiple data* Extensions (SSE) instructions to generate random numbers at almost twice the speed as the original Mersenne twister. AnA-FiTS comes with a distribution of RandomLib [24], a C++ implementation of SFMT. For efficiency, RandomLib initializes an array with random bytes and then transforms the bytes of this array (in steps of 32 or 64 bits) into the required primitive data type (i.e., double, integer, boolean) for the requested range.

For forward simulations, we can exploit two application-specific properties to more efficiently use PRNGs: (i) usually for the ancestry, integer values of less than 4 bytes are required, and (ii) uniform random numbers are frequently drawn from the same interval (e.g., in case of the ancestry from [0,*N*), where *N* is the effective population size of the previous generation). When a large amount of random numbers with these two properties (small integer numbers, constant interval) is required, we can directly initialize a target array from the internal array of the PRNG and transform the minimal number of random bytes for the respective integer type into uniform random numbers for the required range using SSE instructions. For instance, if simulations require sampling 10 integers ∈[ 0,1000), we can copy 20 bytes (=10unsigned short) from the PRNG’s internal array into a location of the ancestry memory block and use vector instructions to transform the 20 bytes into 10 integers of the specified range. Without this optimization, RandomLib would produce 40 bytes of randomness and transform 4 bytes into one integer separately.

#### Non-neutral sequence representation

For forward simulators that work with a given number and pre-defined locations of polymorphic sites (such as [25]), it is straight-forward to represent haplotypes as bit arrays. If we want to simulate a sequence with a huge number of base-pairs (e.g., the human chromosome with 10 Mbp), bit arrays are not the data structure of choice any more because they become too sparse. Thus, in other forward simulator implementations, a haplotype is stored as a sequence of mutations with respect to an initial reference sequence. For instance, SFS_CODE[21] uses self-balancing binary trees to represent the polymorphisms of a haplotype. Sorted binary trees allow for rapid insertion of novel mutations and fast extraction of sub-sequences which are required for creating recombinants.

An alternative data structure is used in forwsim that maintains sorted arrays of sequence positions. Since for AnA-FiTS, we want to store additional information about each mutation (e.g., generation of origin or base), we use a data structure that is based on references/pointers to mutation object instances. When sorted arrays are used, sub-sequence replication for creating recombinants can be efficiently implemented using the memcpy() system call. This is because finding coordinates within the sequence via a binary search has the same time complexity of *O*(*log*(*n*)) as searches in a sorted tree.

Additionally, changing the array size induces a smaller memory allocation overhead than allocating additional nodes in sorted trees. The main drawback of sorted arrays is the insertion cost of O(n). In practice however, obtaining the correct relative position via a binary search is usually more expensive than executing an insertion as long as insertions are implemented via the memcpy() system call. Note that, the memcpy() system call is also vectorized via SSE intrinsics. Finally, the number of insertion operations is relatively small in AnA-FiTS, since we only simulate non-neutral mutations *forward* in time.

We experimented with various sorted tree implementations, for instance, the C++ STL std::set implementation or Judy arrays by [26], which are based on cache-optimized 256-ary trees. For AnA-FiTS, we found that the tree data structures did not outperform our sorted array implementation.

#### Memory management

In forward simulations, the number of distinct haplotype instances containing the sorted array of mutation references and further meta-information and the number of mutation instances changes dynamically at a rapid pace. Thus, dynamic memory allocations (repeated calls to malloc () and free()) that are invoked in the inner parts of nested loops quickly dominate runtimes. As mentioned in the previous section, AnA-FiTS uses block allocation, instead of dynamic allocation whenever possible. Apart from being faster this also prevents heap fragmentation.

One approach to handle the rapid turnover of haplotype and mutation instances more efficiently is to use *free lists* in conjunction with reference counting. The underlying idea is to keep track for each allocated instance, how often it is referenced in the current generation (in case of haplotypes) or in present haplotypes (in case of mutations). Instead of deallocating unreferenced instances, instances are appended to a list, the free list, from which new instances can then be allocated again, when required. However, this allocation strategy still uses more than 10% of total runtime.

**Figure 4:**
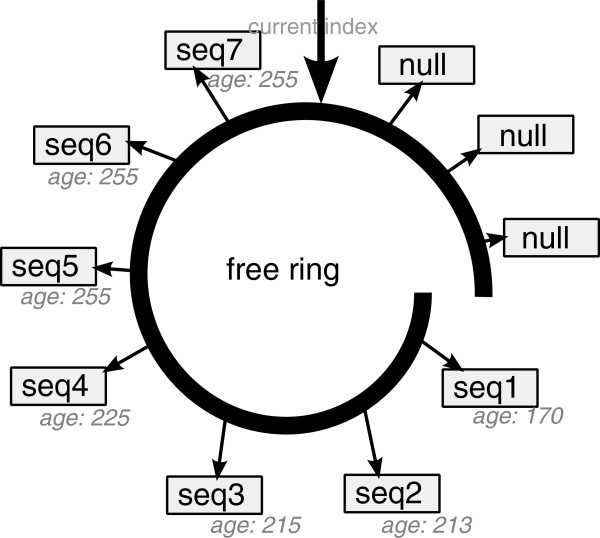
**Free ring.** The allocation structure for haplotype instances (*free ring*). The iterator traverses the ring and returns the first instance that is not present in the current or the previous generation

Instead, we use two task-specific allocation schemes that keep memory allocation overhead to an absolute minimum. For haplotype instances, we deploy a *free ring* scheme (see Figure 4). As the name suggests, a free ring contains references to allocated haplotype instances in a similar way as a free list. Whenever a haplotype is carried over into the next generation, we update its generation number (denoted as *age* in Figure 4). Thus, when haplotype instance memory is requested from the free ring structure, an iterator traverses the elements in the free ring and returns the first instance that is neither used in the current nor the previous generation. If no free instance is available, the free ring capacity is doubled by allocating new haplotype instances. Since it is unlikely that all references of a haplotype will be carried over to the next generation in the sampling phase *without* being subject to recombination or mutation, the iterator typically finds an unused instance after a small number of search steps.

**Figure 5:**
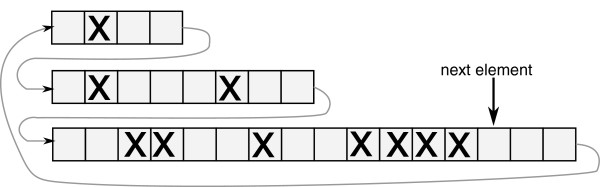
**Reference preserving free ring.** The allocation structure for mutation instances (*address-preserving free ring*). Multiple blocks of mutation instances are allocated. The iterator returns the first instance that is not being used or allocates the next block, if no free instance can be found.

In contrast to this, mutation instances need to be allocated in blocks, since references to instances (as in the free list) represent an inefficient mechanism for this task, because mutation instances are comparably small in size. Moreover, for large numbers of mutations, a corresponding large number of memory allocation invocations would be required, with a negative impact on performance. Allocating monolithic blocks with dynamic re-sizing is also not possible, because addresses of mutation instances can change which would invalidate the references to mutations that are stored in the haplotype instances. Instead, we allocate multiple blocks (extended by a factor of two if the capacity of the previous block is exhausted) of instances and implement an iterator that searches for an unused instance, such that it jumps to the next block when the end of the current block has been reached. When a free instance has been found, it is flagged as *in use*. This flag is updated in a cleanup step that is applied after a fixed number of generations. In this cleanup step, mutation instances that occur in all haplotypes (*fixed* mutations) are removed from all haplotypes. Thus, this phase can be used to efficiently update the *in use* flag of each mutation without inducing a large overhead. Since, under this scheme, the addresses of instances do not change, we refer to this allocation structure as *address-preserving free ring* (see Figure 5).

#### Implementation of neutral sequence extraction

The main challenge in implementing the BEG algorithm is to design an efficient method for extracting sequences from the graph.

For this task, we can use the following property: during the sequence extraction phase, *all* neutral mutations that occurred during simulation are known. Thus, bit arrays are the most adequate data structure for storing neutral sequences. After sorting mutations by their location, each mutation is assigned its corresponding index in the bit array. As a result, the cost of adding a mutation that is encountered during sequence extraction to a given sequence is reduced to merely setting a bit. Handling recombinations is computationally more expensive, since we have to determine the bit array indices of the start and end points for the surviving regions of the parent haplotype via a binary search. Carrying over the relevant mutations from parent haplotypes is implemented as a bit-wise *or*-operation.

This implementation is particularly efficient because the sequence output of the simulation can directly be written as bit array to a binary file. Thus, output files do not require a lot of disk space and programs that directly evaluate the output of a large simulation can efficiently parse and post-process this output data.

## Results and discussion

In this section, we evaluate the runtime performance of AnA-FiTS. In general, conducting a fair comparison between forward simulation tools represents a difficult task, since each simulator uses different assumptions (e.g., finite sites versus infinite sites or pre-defined mutation sites versus random mutation sites). For a meaningful technical analysis, we separately evaluate the two key aspects of AnA-FiTS: efficient implementation of non-neutral forward simulation and ancestry-based simulation of neutral mutations. For realistic use cases, both features independently contribute a certain amount of run time improvement.

For all runs discussed throughout this section, we set mutation and recombination frequencies to *μ* per base=*r*per base=2.5·10^−8^ which roughly correspond to the empirical rates for humans [27,28]. For fixed *μ* and *r*, the runtime depends on the locus length *L* and the effective population size *N*_*e*_. All runs were executed on a 4×8 core machine (AMD Opteron 6174) with a total of 256 GB RAM. The execution times provided here are the result of 4 independent runs with different random number seeds. We discard the slowest runtime in order to correct for the impact of seed values that generate exceptionally expensive simulations and average over the remaining three.

Moreover, we validate AnA-Fits by comparing the output of our software to datasets generated with established simulation codes. Finally, we describe runtime gains for the simulation of partially neutral sequences, specifically in a whole-genome setting for human sequences.

### Runtime improvement: forward simulation only

Here, we evaluate the efficiency of the pure forward simulation part of our implementation and compare it to SFS_CODE. AnA-FiTS can simulate each mutation forward in time by choosing a constant small selection coefficient *s*≠0 for each mutation that is to be simulated. If *s* is small enough, it is effectively neutral. This way the BEG algorithm is not applied. Simulating close to neutral mutations (*s*:=0), forward in time, represents the most expensive case, since for large values of *s* the mutation quickly undergoes selection and for small negative values it is removed from the gene pool by selection. In both cases the mutation quickly vanishes which in turn reduces runtimes.

**Figure 6:**
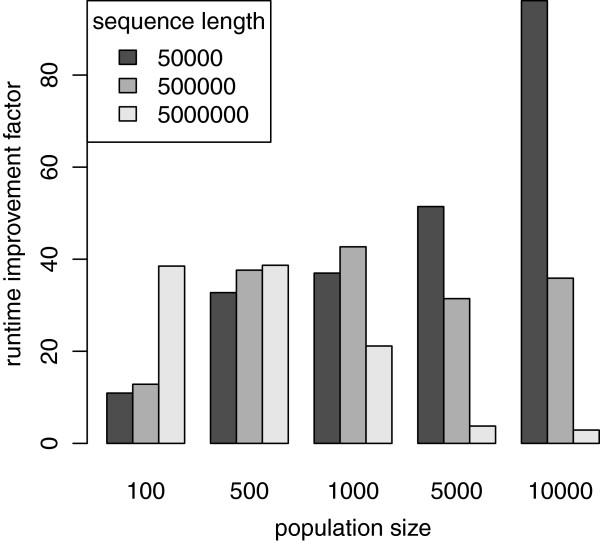
**Performance comparison to SFS_CODE**. Runtime comparison between SFS_CODE and AnA-FiTS without the BEG algorithm. *y*-axis depicts runtime improvement factor (SFS_CODE runtime divided by AnA-FiTS runtime).

In runs with SFS_CODE, we simulate neutral mutations forward in time. In general, it is not possible to efficiently simulate one contiguous locus of the length of a chromosome with SFS_CODE (i.e., this is orders of magnitude slower). Instead, for simulating a locus of 1,000,000 base pairs (bp), the user has to break up the locus into a number of linked loci (e.g., 100 × 10,000). For obtaining SFS_CODE runtimes, we did not optimize the break-up strategy exhaustively, but followed the general suggestions by the author and simulated chromosome lengths listed in Figure 6 as 50·1 Kbp, 50·10 Kbp and 500·10 Kbp.

Figure 6 depicts the relative runtimes of both programs. Overall, AnA-FiTS is between 2.9 and 96.2 times faster than SFS_CODE. In all except 2 runs, AnA-FiTS is at least one order of magnitude faster than SFS_CODE.

The runtime improvements do not show a clear dependency on one of the two input dimensions (*N*_*e*_, sequence length). We assume that this is because, for certain steps, SFS_CODE switches between alternative implementations depending on the number of recombination/mutation events that are expected per generation. Further parameters that may influence the runtime differences between the codes are: (i) frequency of the cleanup steps in either code (fixed to 100 in AnA-FiTS since in AnA-FiTS no significant performance impact could be detected) and (ii) number of linked loci the sequence was split into (SFS_CODE only).

### Runtime improvement: ancestry-based simulation

As already mentioned, the BEG algorithm is inspired by the postponed simulation of neutral mutations as implemented in forwsim. Thus, in order to evaluate the stand-alone efficiency of the BEG algorithm, we compare the runtime for simulating entirely neutral sequences between AnA-FiTS and forwsim. Note that, forwsim simplifies the Fisher-Wright model in two ways: (i) while it assumes finite sites, at most one mutation per site is allowed and (ii) haplotypes undergo at most one recombination event per generation. We omit a comparison between forwsim and AnA-FiTS that includes forward simulation of non-neutral mutations. The reason for this is that, forwsim assumes a fitness model that differs from the model implemented in AnA-FiTS and SFS_CODE: for each non-neutral site, the user has to specify selection coefficients for the two homozygous cases and the heterozygous case. Thus, forwsim needs to iterate over all non-neutral mutations of each individual and recompute the fitness for each individual as opposed to AnA-FiTS and SFS_CODE, where one simply multiplies the per-haplotype coefficients. In the final analysis, the non-neutral mutation model implemented in forwsim, is more powerful (balancing selection can be accommodated), but slower and has a hard limit with respect to the number of non-neutral mutations that can be simulated. In AnA-FiTS, the fitness of an individual is the product of the fitness effects *f* (where *f*=1−*s* with selection coefficient *s*) of all mutations on either haplotype.

**Figure 7:**
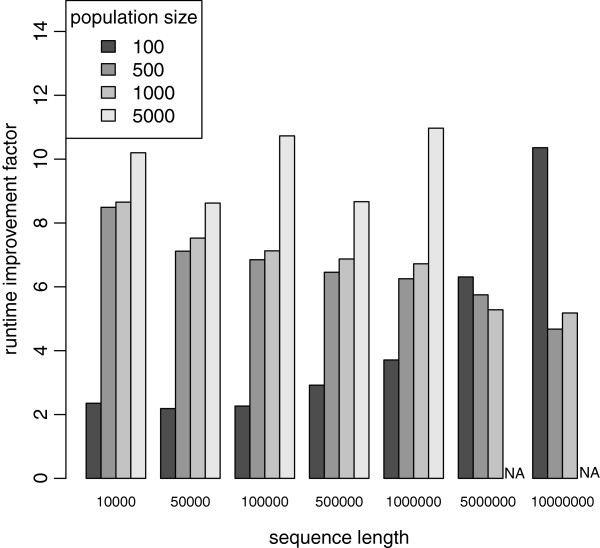
**Performance comparison to forwsim**. Runtime comparison between forwsim and AnA-FiTS for simulation of strictly neutral mutations. *y*-axis depicts runtime improvement factor (forwsim runtime divided by AnA-FiTS runtime). Missing bars (*NA*) indicate prohibitive runtime.

Figure 7 depicts the relative runtimes of forwsim and AnA-FiTS. The BEG algorithm allows for simulation of neutral sequences that is between 2.2 and 11.0 faster than the delayed simulation in forwsim. In less than 25% of all cases (all *N*_*e*_=100), the runtime improvement is below a factor of 5. Since for these cases only a small number of recombination/mutation events occur along short sequences and a small effective population size of 100 is used, the runtime improvement in AnA-FiTS is mainly due to the more efficient random number generation. For longer sequences with a higher number of individuals, our BEG algorithm is substantially more efficient than the postponing algorithm of forwsim. Note that, these runtimes already include the overhead that is associated with generating and writing the BEG to file.

### Validation

We performed a validation analogous to the validation of SFS_CODE[29]. Since under neutrality, summary statistics of datasets that are simulated using AnA-FiTS can easily be compared with the equivalent summary statistics generated by the popular coalescent software ms[12], we mainly validate datasets that have been simulated under neutrality. We also compare simulations with AnA-FiTS under selection to datasets produced by SFS_CODE.

#### Under neutrality

To ensure that population genetic simulation software produces correct results, it is common to compare summary statistics for datasets produced by the software that needs to be validated to the respective summary statistics of widely-used and well-tested software packages. To date, ms is among the most cited population genetic simulation tools. Slight deviations in summary statistics may be expected, since for instance ms implements an infinite-sites model of mutation, whereas AnA-FiTS implements a full finite-sites model. We used AnA-FiTS to simulate chromosome-sized sequences. Thus, the effect of non-single nucleotide polymorphisms or multiple mutation events that give rise to the same genotype should not influence the respective summary statistics.

In addition to this standard validation setup, for AnA-FiTS we also have to show that (i) the BEG-algorithm yields correct results (which could be shown by a formal mathematical proof alternatively) and that (ii) the algorithm is correctly implemented. Thus, irrespective of the respective simulation scenarios we show, that all three simulation modes produce the same summary statistics as ms: (i) all mutations are simulated forward in time (referred to as *AF-FOR*), (ii) a part of the mutations is simulated forward in time (*s*:=0), while the rest is obtained via an ancestry-based simulation (referred to as *AF-MIX*), (iii) all mutations are simulated via ancestry-based simulation (referred to as *AF-ANC*),

We test the correctness of our algorithm and implementation for 5 specific scenarios employing the *number of haplotypes*, the *number of segregating sites*, the *nucleotide diversity*, and the *site frequency spectrum* as summary statistics. The scenarios are (for a present population size of *N*_*p*_): (i) mutation only (no recombination), (ii) mutation and recombination, (iii) mutation, recombination, a doubling in population size taking place 0.2·*N*_*p*_ generations ago, (iv) mutation, recombination, a with a factor-2-bottleneck taking place 0.25·*N*_*p*_ generations ago, (v) mutation, recombination, with an exponential population growth setting in 0.5·*N*_*p*_ generations ago.

**Figure 8:**
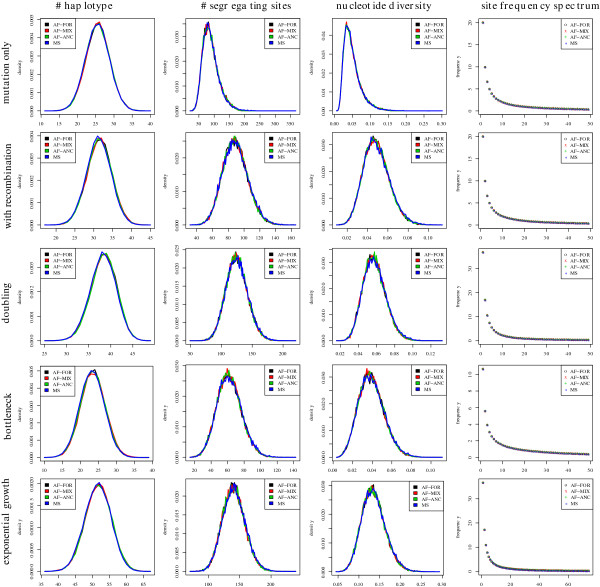
**Verification under neutrality.** Summary statistics (one per column) for datasets created with three different parameter settings of AnA-FiTS (see text) or with ms for reference. Each row corresponds to one of the 5 distinct simulation scenarios (see text).

For all scenarios, we assume an initial population size of *N*_*e*_:=500, a sequence length *L*:=10^6^, a mutation rate *m*:=10^−8^ and a recombination rate *r*:=10^−8^ (where applicable). For the forward simulation scenario we calculated 10·*N*_*e*_ generations in total. For each scenario, the sampling size was set to 50 sequences, except in the exponential scenario, where we sampled 75 sequences. Figure 8 shows the distribution of summary statistics after 30,000 simulations for each of the three AnA-FiTS parameters and for corresponding ms runs. To visualize the number of segregating sites and the nucleotide density, we used the Freedman-Diaconis’ rule [30] for a binning that retains a high level of resolution. Even using such a high resolution, the distributions in Figure 8 almost correspond perfectly, with the exception of minor range deviations around the mode of the distribution.

#### Under selection

To validate our software for simulations with selection, we compared the same summary statistics (see previous section) as obtained by AnA-FiTS to summary statistics for datasets simulated with SFS_CODE. To keep the computational effort within reasonable limits, we reduced the number of simulations to 15,000, the sequence length to 10^5^ bp, the effective population size to *N*_*e*_=250, and set the mutation and recombination rate per sequence *and* per base pair to *m*:=*r*:=10^−6^ (sampling size retained at 50 sequences).

**Figure 9:**
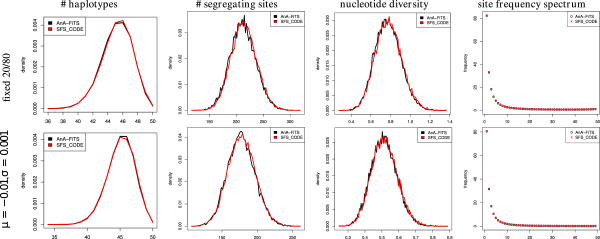
**Verification under Selection.** Summary statistics (one per column) for datasets created either with AnA-FiTS (black) or with SFS_CODE) (red). Columns correspond to the respective summary statistic and rows to simulation scenarios that are described in the text.

Figure 9 shows a comparison of summary statistics for two scenarios. In scenario one, we chose a fixed selection coefficient *s*:=0.01 using either a positive (20% of all mutations) or a deleterious effect (80% of mutations) for all mutations. In scenario 2, selection coefficients are drawn from a normal distribution with mean *μ*=−0.01 (i.e., mutations will have a deleterious effect) and standard deviation *σ*=0.001. This particularly high value for the mean and the fixed value of selection coefficients respectively was chosen to ensure that selection does indeed affect the summary statistics that are being assessed.

For SFS_CODE simulations, we simulated the 100 Kbp sequence as 20 linked loci of length 5 Kbp. As shown in the Figure 9, the distributions of summary statistics of AnA-FiTS show a high level of agreement to the respective distributions obtained with SFS_CODE.

### Large-scale simulation of partially neutral sequences

Our algorithmic techniques and low-level technical optimizations improve AnA-FiTS execution times by one to two orders of magnitude compared to SFS_CODE for plain forward simulation of sequences. If the user specifies a fraction of mutations to be neutral, AnA-FiTS can become faster by more than one *additional* order of magnitude (see Table 1).

**Table 1:** Performance gains with ancestry-based simulation

	***N***_***e ***_**= 1,000;*L *= 5·10**^**6**^	***N***_***e ***_**= 5,000;*L *= 10**^**6**^
AF, *F*=100*%*	20.2 secs	128.9 secs
AF, *F*=10*%*	3.0 secs	32.6 secs
AF, *F*=1*%*	2.0 secs	26.6 secs
AF, *F*=0*%*	1.3 secs	16.5 secs
SFS_CODE	427.3 secs	2910.4 secs

AnA-FiTS (AF) runtimes (in *sec*) for simulation of neutral sequences with a varying proportion *F* of mutations simulated forward in time (e.g., *F*:=10*%*⇒ 10% of mutations are simulated forward in time, 90% using the BEG algorithm); SFS_CODE runtime for comparison. Simulations for 10·*N*_*e*_ generations, with per-base recombination and mutation frequency of 2.5·10^−8^.

Given these runtime improvements, coupled with the continuous advances in hardware, large-scale simulations of genomic regions at the chromosome level are becoming feasible. AnA-FiTS also allows for simulating multiple chromosomes (resp. large unlinked loci, one separate graph is generated per locus). If we reduce the fraction of non-neutral mutations to 10%, the entire (female) human genome (simulated as 23 unlinked loci with a total 3.037 ·10^9^ bp for 10·*N*_*e*_ generations) can be simulated for 100 individuals within 55.2 seconds, resulting in a total of 159,651 segregating sites. For 200 individuals (same parameters), our implementation requires 441.7 seconds, yielding a dataset with 323,673 segregating sites.

## Conclusions

We discussed the design and optimization of a highly efficient simulator for the forward-in-time simulation of population genetic datasets. Our software is up to two orders of magnitude faster than competing state-of-the art software. Moreover, we described the BEG algorithm, that may further accelerate simulation for the common scenario, when a fraction of mutations does not have an impact on the fitness of individuals. Our algorithm outperforms a similar exploratory approach by up to one order of magnitude, while it is at the same time capable of retaining the entire observable history for each haplotype. We demonstrated that our software allows for forward-in-time simulation at the whole-chromosome and even whole-genome level within acceptable execution times.

Our novel graph structure (the BEG) may become useful for population geneticists. Graph metrics can be computed on this structure, and the surviving material of any ancestrial haplotype can be reconstructed for any given point in time. Alternatively, local trees could be extracted from the BEG for each sequence segment that did not undergo recombination.

We believe that the availability of AnA-FiTS will lead to an increased usage of forward simulation in population genetics, given its importance for research on natural selection, and in the context of approximate Bayesian computation. Thus, our tool can handle the analytical challenges of the post-genomic era.

## Abbreviations

PRNG: Pseudo random number Generator.

## Competing interests

The authors declare that they have no competing interests.

## Authors’ contributions

AJA and AS designed the study. AJA developed the algorithm and implemented/optimized the simulator. AJA and AS wrote the paper. Both authors read and approved the final manuscript.
